# THE IMPACT OF SOCIAL WORKERS ON POST-ACUTE CARE DISCHARGE OUTCOMES

**DOI:** 10.1093/geroni/igz038.1309

**Published:** 2019-11-08

**Authors:** Amy Restorick Roberts, Amy Restorick Roberts, John R Bowblis, Austin C Smith

**Affiliations:** Miami University, Oxford, Ohio, United States

## Abstract

Background: Social service staff may play a key role in helping post-acute care patients in skilled nursing facilities return home, yet few studies quantify how social service staff contribute to better patient outcomes. Method: A quasi-experimental statistical approach, regression discontinuity, was used among newly-admitted, Medicare post-acute care patients (65+) to examine the relationship between higher qualifications of social service workers and various discharge outcomes. National data (2011-2015) were drawn from the Online Survey Certification and Reporting system, the Certification and Survey Provider Enhanced Reports, and the Minimum Data Set. Findings: Patients in facilities with a greater proportion of more qualified social service staff (qualified social workers vs. paraprofessionals) had better discharge outcomes. Post-acute care patients were more likely to be discharged home within 30 days, compared to being re-hospitalized or remaining in the facility. Conclusion: Policymakers and providers should support efforts to increase the qualifications of social service staff.

